# Dietary intake and breast cancer risk in black South African women: the South African Breast Cancer study

**DOI:** 10.1017/S0007114518003744

**Published:** 2019-02-01

**Authors:** Inarie Jacobs, Christine Taljaard-Krugell, Cristian Ricci, Hester Vorster, Sabina Rinaldi, Herbert Cubasch, Ria Laubscher, Maureen Joffe, Tertia van Zyl, Shane A. Norris, Isabelle Romieu

**Affiliations:** 1 Centre of Excellence for Nutrition, North-West University, Private Bag X6001, Potchefstroom 2520, South Africa; 2 International Agency for Research on Cancer, Section of Nutrition and Metabolism, 150 cours Albert Thomas, 69372 Lyon CEDEX 08, France; 3 Department of Surgery, Faculty of Health Sciences, University of Witwatersrand, Private Bag X2600, Houghton, Johannesburg 2041, South Africa; 4 Non-Communicable Diseases Research Division, Wits Health Consortium (PTY) Ltd, Parktown, Johannesburg 2193, South Africa; 5 South African Medical Research Council, PO Box 19070, Tygerberg, Cape Town 7505, South Africa; 6 MRC Developmental Pathways to Health Research Unit, Department of Paediatrics, Faculty of Health Sciences, University of Witwatersrand, Private Bag X3, Johannesburg 2050, South Africa; 7 Centre for Research on Population Health, National Institute of Public Health, CP 62100, Cuernavaca, Morelos, Mexico; 8 Hubert Department of Global Health, Emory University, Atlanta, GA 30329, USA

**Keywords:** Dietary intake, Black women, Breast cancer, South Africa

## Abstract

Incidence rates of breast cancer (BC) are increasing in South Africa. The aim of this study was to investigate the association between dietary intake and BC risk in black South African women. The study population included 396 BC cases and 396 population-based controls matched on age and residence, participating in the South African Breast Cancer study. Diet was assessed using a validated quantified FFQ from which twelve energy-adjusted food groups were formed and analysed. OR were estimated using conditional logistic regressions, adjusted for confounding factors, comparing highest *v.* lowest median intake. Fresh fruit consumption showed an inverse association with BC risk (OR=0·3, 95 % CI 0·12, 0·80) in premenopausal women, whilst red and organ meat consumption showed an overall inverse association with BC risk (OR=0·6, 95 % CI 0·49, 0·94 and OR=0·6, 95 % CI 0·47, 0·91). Savoury food consumption (sauces, soups and snacks) were positively associated with BC risk in postmenopausal women (OR=2·1, 95 % CI 1·15, 4·07). Oestrogen receptor-positive stratification showed an inverse association with BC risk and consumption of nuts and seeds (OR=0·2, 95 % CI 0·58, 0·86). Based on these results, it is recommended that black South African women follow a diet with more fruit and vegetables together with a decreased consumption of less energy-dense, micronutrient-poor foods such as savoury foods. More research is necessary to investigate the association between BC risk and red and organ meat consumption. Affordable and practical methods regarding these recommendations should be implemented within health intervention strategies.

Breast cancer (BC) is currently the most general cancer diagnosed in women and the second leading cause of cancer mortality globally^(^ ^1^ ^)^. Increased incidence rates in low-income and middle-income countries like South Africa are predicted in forthcoming years^(^ ^2^ ^)^. Modifiable lifestyle risk factors such as physical activity, body weight and dietary intake are key factors influencing BC risk^(^ ^3^ ^,^ ^4^ ^)^.

The extent of dietary factors contributing to BC risk is not yet fully known. However, the WHO previously estimated that 30–50 % of all cancer cases could be prevented by avoiding a combination of risk factors including dietary factors^(^ ^5^ ^)^. Dietary intake in different population groups across South Africa has extensively been studied by various authors^(^ ^6^ ^–^ ^11^ ^)^. Hence, valuable insights into South African dietary intake were obtained for health promotion interventions. The link, however, between dietary intake and BC risk within black South African women has not been given sufficient attention. Research investigating this specific association is lacking in South Africa. Therefore, evidence for guidelines towards a population-specific diet to prevent BC is absent.

Prevention of BC would be the most cost-effective strategy for decreasing cancer incidence rates in a low- to middle-income country like South Africa. Currently, modification towards a healthier diet (nutrient-rich and less energy-dense foods) is encouraged by the World Cancer Research Fund (WCRF) to promote prevention of various cancers^(^ ^12^ ^)^. In line with the WCRF, the South African Food Based Dietary Guidelines (SAFBDG) also advises South Africans to follow a healthier diet as a strategy to reduce non-communicable diseases like obesity and cancer^(^ ^13^ ^)^.

Despite the promotion of a less energy-dense and more nutrient-rich diet, a more Westernised diet is ever increasingly being followed by black South African women^(^ ^14^ ^)^. A Westernised diet is defined by high intakes of energy-dense foods such as refined grains, processed meats, added sugar and saturated fatty foods^(^ ^15^ ^)^ and is frequently associated with a monotonous diet in South Africa. Monotonous diets are often a result of food insecurity and poverty that contributes to increased consumption of low-cost, energy-dense foods^(^ ^14^ ^)^. Increased consumption of energy-dense foods might increase the risk of obesity. The continuous update project (CUP) report of BC risk and prevention acknowledges that obesity increases BC risk in postmenopausal women^(^ ^4^ ^)^. Thus, overconsumption of high energy-dense foods and low physical activity levels together with increasing obesity rates in the black female population of South Africa^(^ ^14^ ^,^ ^15^ ^)^ is worrisome risk factors for BC and raises concerns for health prevention strategies.

The aim of this study was to determine the association between dietary intake and BC risk in a population-based study of black South African women within the SABC case–control study.

## Methods

### Study population

The SABC study is a population-based, case–control study conducted on black South African women from the greater Soweto population in Gauteng, South Africa.

Case participants (*n* 396) were women over the age of 18 years, with primary first, invasive, pathologically confirmed BC diagnosed at the Chris Hani Baragwanath Breast Unit in Soweto. Case participants were recruited, before any treatment, from December 2014 until June 2017. Control participants (*n* 396) were healthy, non-blood relatives of case participants matched by age (±5 years), who lived in the same neighbourhood as the cases, with no history of cancer diagnosis. Control participants were not matched on any known BC risk factors. The sample size was sufficient to obtain a power of 90 % (type II error rate *β*=10 %) for OR ≥1·3 when type I error was set to 5 %.

### Dietary assessment

A validated and reproducible culture-specific quantified FFQ (QFFQ) was used in combination with food portion pictures, household utensils and food models together with the South African Food Composition Tables to determine dietary intake^(^ ^16^ ^–^ ^19^ ^)^. The QFFQ included 145 food items grouped together from the most frequently consumed staple foods to those foods consumed in small amounts. The food portion picture booklet comprised life-size colour photographs of thirty-seven foods in three portion sizes and photographs of utensils to estimate the portion sizes^(^ ^20^ ^)^. Women were asked about their intake over the past month to estimate their habitual dietary intake. The frequency included the number of times per d, per week, per month or seldom. The South African Food Composition Tables were used to code and calculate the dietary intake from the frequency and portion size reported on the QFFQ^(^ ^19^ ^)^. Household measurements were converted to grams by means of standardised tables^(^ ^21^ ^)^. The effects of seasonality were addressed by measuring dietary intakes throughout the year in all seasons.

### Non-dietary assessments

Face-to-face interviews were conducted by trained fieldworkers and investigators. Self-reported demographics and socio-economic indicators such as level of education and income/month were obtained. Detailed information were collected regarding ethnicity, history of health, family history of BC, reproductive risk factors (age at menarche and at menopause for postmenopausal women only, age/year at each full term pregnancy, and its outcome, breast-feeding history at for each live birth, use of oral contraceptives and hormone replacement therapy, family history of cancer, breast health (previous breast lumps by breast laterality and breast pains), smoking habits and physical activity (household and recreational). Anthropometric measurements (weight, height, sitting height and waist circumference) were collected using standardised procedures accredited by Lohman’s laws^(^ ^22^ ^)^. BMI was calculated using measured height and weight (kg/m^2^). Questionnaires used to obtain above mentioned information were validated and proven to be reproducible in studies conducted in South Africa^(^ ^23^ ^,^ ^24^ ^)^.

### Ethical approval

Ethical approval for the SABC study was granted by the International Agency for Research on Cancer and by the University of the Witwatersrand Committee for Research on Human Subjects (ethical no. M140980). Permission to conduct research at Chris Hani Baragwanath Academic Hospital was obtained from the Gauteng Province Medical Advisory Committee.

This single dietary study obtained ethical approval from the Human Research and Ethics Committee of the North-West University (NWU-00118-17-S1). Ethical approval was also granted for the use of the validated Prospective Urban and Rural Epidemiological QFFQ^(^ ^18^ ^)^. All subjects gave written informed consent prior participation.

### Statistical analysis

A total of 792 black female participants (396 cases and 396 controls) could be matched from the original 874 enroled participants (415 cases and 459 controls). Unmatched case and control participants were due to missing dietary data, incorrect data captured, unmatched geographical areas and withdrawal of participants. Baseline characteristics were described for BC cases and healthy controls. Normally distributed variables were presented as means and standard deviations, whilst variables with a skew distribution were presented as median, upper and lower quartiles. Categorical variables were presented as frequencies and percentages. Mean differences in normally distributed data between cases and controls were estimated using Student’s *t* test for independent samples, while skewed variables were tested by the Mann–Whitney *U* test. Categorical variables were compared using Pearson’s *χ* ^2^ test.

Energy-adjusted intake was used for analyses due to individuals whom generally alter their intake of nutrients and foods primarily by changing the composition of their diets, rather than the total amount consumed^(^ ^25^ ^)^.

Dietary intake obtained from the QFFQ was divided into twelve food groups: cooked porridge (maize meal, oats, maltabella), starchy grains (breakfast cereals, pasta, bread, rice, cake flour, starchy vegetables), non-starchy vegetables (all other vegetables), fresh fruit, legumes (soya and beans), nuts and seeds, milk and milk products, animal protein, fats and oils (monounsaturated, polyunsaturated fat and saturated fats), added sugar (sweets, sugary drinks, jam and pudding), savoury snacks (sauces, potato crisps, spices, soups) and alcohol. Animal protein were analysed separately from its original compilation to estimate the association with BC risk and different animal proteins. The following subgroups were created from the animal protein food group: red meat (mutton, beef and stews), organ meat (liver and curried offal), chicken (offal, liver, kidneys and all other chicken meat), eggs (chicken eggs, fried, scrambled and poached), processed meat (ham, sausages and polony) and fish (hake, fish fingers and pilchards). Red meat is usually classified as mutton, beef, lamb, goat and pork including offal/organ meat thereof. Due to the differences in energy and nutrients, organ meat was separated from red meat in this sample. Missing information regarding food intakes was imputed using the expectation–maximisation algorithm before performing analysis^(^ ^26^ ^)^. A generalised linear model was used to estimate the differences in least square means measured in kJ of individual food groups (continuous variable) between cases and controls. The effect of potential confounders was tested by including additional variables into the generalised linear and conditional logistic regression models. The following confounders were examined: ethnicity (Zulu/Pedi/Swazi, Xhosa, Sotho, Tshwane, Venda, Tsonga and Ndebele), individual income (R1-R3000, R3001-R6000 and R6001+), level of education (none/primary school, high school and college/postgraduate/diploma), smoking (smokers and non-smokers), waist circumference (continuous data), habitual physical activity/d (active and less active), age at menarche (<15 *v.* >15 years of age), full-term pregnancy (yes/no), age at first pregnancy (<24 *v.* >24 years of age), age of menopause (<48 *v.* >48 years of age), parity (≤3 children *v.* >3 children), ever breast-feeding (yes/no), duration of breast-feeding (months), use of exogenous hormones (hormonal birth control to avoid pregnancy (oral contraceptives and injections)), or hormone replacement therapy/combined hormone replacement therapy after menopause, family history of BC (yes/no). Only menopausal status, ethnicity, waist circumference, physical activity, level of education, income/month, use of birth control, ever breast-feeding, age at menarche, age of menopause and onset and family history of BC influenced crude analysis by more than 10 %. All the remaining food groups not used as the independent variable were included in the regression model to adjust for confounding effects since food groups are not eaten in isolation.

Conditional logistic regression was applied to estimate OR and 95 % CI to measure the risk of BC in relation to highest *v.* lowest energy (kJ) intake (determined by median intake) of food groups. Adjustments for possible confounding factors were made in a sequential model. Unadjusted estimates between matched case and control participants were reported in model A, whilst model B adjusted for the same confounding factors used in the generalised linear model. Analyses were also stratified according to menopausal status, oestrogen receptor-positive (ER^+^) and oestrogen receptor-negative (ER^–^) tumour types.

## Results

The distribution of selected characteristics amongst cases and controls are reported in Table 1. As expected from the matched design, age was similar amongst cases and controls (54·68 (sd 12·94), 54·70 (sd 12·90) years) and ranged from 26 to 88 years. Weight and BMI had a similar distribution between case and control participants.

**Table 1:** Distribution of baseline characteristics between cases and controls* (Numbers and percentages; mean values and standard deviations; medians and 25th and 75th percentiles)

	Controls (*n* 396)				Cases (*n* 396)			
Characteristics	*n*			%	*n*		%	*P*
Age (years)								0·9831
Mean	54·6				54·7			
sd	12·9				12·9			
Weight (kg)								0·2660
Mean	78·9				77·5			
sd	17·7				17·6			
Height (cm)								0·3427
Mean	157·9				157·5			
sd	6·3				6·4			
BMI (kg/m^2^)								0·3090
Mean	31·8				31·4			
sd	6·9				7·0			
Underweight	5			1·3	11		2·8	
Healthy BMI	63			15·9	71		17·9	
Overweight BMI	93			23·5	87		22·0	
Obese BMI	235			59·3	227		57·3	
WC (cm)								0·0113
Mean	95·8				93·3			
sd	13·7				13·8			
TE (kJ/d)								0·2631
Median	8990				9146			
25th, 75th percentiles	7184, 10284				6812, 9759			
Protein (g/d)								0·0831
Median	63·5				63·8			
25th, 75th percentiles	49·2, 93·1				47·4, 82·7			
Percentage of TE				12·0			11·8	
Animal protein (g/d)								0·0057
Median	34·1				31·0			
25th, 75th percentiles	22·9, 48·7				20·6, 45·1			
Plant protein (g/d)								0·9242
Median	29·5				29·6			
25th, 75th percentiles	22·5, 40·2				22·6, 39·7			
Fat (g/d)								0·1373
Median	64·4				64·8			
25th, 75th percentiles	47·2, 95·7				42·4, 91·9			
Percentage of TE				27·2		26·9		
Saturated fat (g/d)								0·0499
Median	19·1				17·9			
25th, 75th percentiles	12·6, 27·8				11·4, 26·1			
MUFA (g/d)								0·0479
Median	20·6				20·5			
25th, 75th percentiles	14·3, 31·7				12·3, 28·3			
PUFA (g/d)								0·3934
Median	17·5				17·2			
25th, 75th percentiles	11·70, 26·73				11·1, 25·4			
CHO (g/d)								0·4412
Mean	338·7				330·8			
sd	147·3				143·5			
Percentage of TE				64·0			61·4	
Added sugar (g/d)								0·3400
Median	67·9				65·3			
25th, 75th percentiles	39·9, 109·7				38·4, 105·5			
Percentage of TE				12·0			12·1	
PA (MET/week)								0·1418
Median	110·2				114·0			
25th, 75th percentiles	81·6, 149·7				82·8, 163·1			
Energy from alcohol (kJ/d)								0·2007
Median	312				79			
25th, 75th percentiles	288, 2204				29, 1954			
Percentage of alcohol contribution to TE				3·4			0·8	
Ethnicity								
Zulu and Pedi	26			6·5	25		6·4	
Xhosa	22			5·5	22		5·6	
Sotho	108			27·4	144		36·4	
Tswana	19			4·8	19		4·8	
Venda	40			10·1	56		14·1	
Tsonga	65			16·4	35		8·8	
Ndebele	116			29·3	95		23·9	0·004
Smoking	44			11·1	35		8·8	0·286
Level of education								
None/primary	71			18·0	97		24·5	
High school	279			70·5	257		64·9	
College/university/postgraduate	46			11·6	42		10·6	0·078
Individual income/month								
R1–R3000	335			84·6	344		86·9	
R3001–R6000+	61			8·5	52		8·6	0·364
Ever pregnant	382			96·5	377		95·2	0·374
Number of children (children)								0·3739
Median	3				3			
25th, 75th percentiles	2, 4				2, 4			
Age at first pregnancy, *n*/*N* (%)	24·5/32			19·5, 26	23·5/26		19, 28	0·567
Full-term pregnancy in parous women	382			100	377		100	0·3739
Ever breastfed, *n*/*N* (%)	349/382			91·3	339/377		89·9	0·293
Duration of breast-feeding† (months)								0·1868
Median	32				30			
25th, 75th percentiles	12, 60				8, 58			
Use of birth control (contraceptives)								0·355
Mean	215				229			
sd	54·3				57·8			
Stage at BC diagnoses								
I					24	6·5		
II					175	44·8		
III					161	40·8	
IV					31	7·9		
BC subtype								
ER^+^					312	78·8		
PR^+^					281	70·1		
HER2					114	28·8		
Receptor status								
HER2 enriched					21	5·3		
Luminal A					40	10·1		
Luminal B					269	67·9		
TNBC					66	16·7		
Menopause status								
Premenopausal	137			34·6	140		35·4	
Postmenopausal	259			65·4	256		64·6	0·584
Age at menopause‡ (years)								0·7899
Median	48				47			
25th, 75th percentiles	44, 50				42, 50			
Family history of BC	17			4·3	25		6·3	0·2046
Age at menarche (years)								0·2485
Median	15				15			
25th, 75th percentiles	13, 16				13, 16			
≤15	182			45·9	169		42·6	
>15	214			54·1	227		51·4	0·9407
Use of HRT§	2			0·7	2		0·7	0·134

WC, waist circumference; TE, total energy; CHO, carbohydrates; PA, physical activity; MET, metabolic equivalents of task; BC, breast cancer; ER^+^, oestrogen receptor positive; PR^+^, progesterone receptor positive; HER2, human-epidermal growth factor-2; TNBC, triple negative breast cancer; HRT, hormone replacement therapy.

* Student’s *t* test for independent variables, Mann–Whitney *U* test for skewed data and Pearson’s χ^2^ test for categorical variables

† In breast-feeding women only.

‡ Among postmenopausal women only.

§ In postmenopausal women only.

More than 80 % of the study sample, cases (80·0 %) and controls (82·3 %), were either overweight or obese. High total energy intake/d was reported in both cases and controls, with a median of 8990 (25th, 75th percentiles 7184, 10 284) kJ in controls and 9142 (25th, 75th percentiles 6812, 9759) kJ in cases. In addition, low physical activity levels with little variation were noted. Neither cases nor controls’ total weekly physical activity levels adhered to the recommended 600 metabolic equivalents of task (MET)/week^(^ ^4^ ^)^. In case participants, ER^+^ tumour type together with receptor type luminal B were most prominent. Triple negative breast cancer, the most aggressive BC tumour type, accounted for 16·7 % of case participants.

Compared with controls, cases had a significant smaller waist circumference and lower animal protein, saturated fat and mono-unsaturated fat intake. Comparison between cases and controls differed significantly in ethnicity. Zulu-, Pedi-, Xhosa- and Tswana-speaking participants were evenly distributed amongst cases and controls. More Sotho- and Venda-speaking participants were noted in the case group, whilst more Ndebele- and Tsonga-speaking participants were noted in the control group. No significant differences were observed in the distribution of other macronutrients, level of education, individual income, menopausal status and smoking between cases and controls.

Differences in mean energy (kJ) intake (adjusted for confounders mentioned above) between cases and controls in food groups are reported in Table 2. Significant differences between cases and controls were observed in all food groups except cooked porridge. The control group reported higher energy intakes in all food groups except cooked porridge.

**Table 2:** Adjusted means of dietary factors for cases (*n* 396) and controls (*n* 396) (Least square means with their standard errors)

	Controls*		Cases		
	Mean (kJ)	se (kJ)	Mean (kJ)	se (kJ)	*P* for difference†
Cooked porridge	1842	90·9	1815	92·3	0·768
Starchy grains	3155	162·4	2482	163·9	<0·001
Vegetables	667	54·5	516	54·6	0·023
Fresh fruit	949	61·5	747	61·5	0·007
Legumes	215	21·6	133	21·7	0·004
Nuts and seeds	889	101·1	632	101·1	0·035
Milk and milk products	573	40·9	343	41·4	<0·001
Animal protein	4551	328·7	3234	328·7	<0·001
Fats and oils	1737	164·1	1134	165·3	<0·001
Sugar	3009	214·6	2136	214·1	<0·001
Savoury snacks	2064	203·0	1398	205·4	<0·001

* Reference group.

† Adjusted for menopausal status, ethnicity, waist circumference, physical activity, level of education, income/month, use of birth control (hormonal/oral contraceptives), ethnicity, ever breast-feeding, age at menarche, age of menopause onset and family history of breast cancer.

For the purpose of this study and using the SAFBDG, vegetables, milk and milk products, legumes, fresh fruit, nuts and seeds were classified as less energy-dense food groups, whilst starchy grains, savoury foods, animal protein (high fat content), cooked porridge and sugar were considered to be more energy-dense food groups. Online Supplementary Fig. S1 presents the percentage distribution of median energy intake/d of food groups in case and control participants. Food groups that are likely to be more energy dense contributed to more than 75 % of the total energy intake in both case and control participants. Less energy dense food groups accounted for 18·4 % of the total energy intake in controls and 14 % in cases.

A total of 196/792 participants consumed alcohol in this sample, whilst non-consumers accounted for 80·8 % in case and 69·7 % in control participants and was therefore excluded as a food group in the analysis. The animal protein food group consisted of red meat, organ and offal meat, fish, chicken, eggs and processed meat. The savoury snacks food group consisted out of soup powders, spices, potato crisps, sauces and salt biscuits.

The association between dietary intake and BC risk is reported in Table 3. After adjusting for confounding factors, inverse associations with BC risk were noted with fresh fruit consumption overall and especially in premenopausal women (OR=0·6, 95 % CI 0·43, 0·94 and OR=0·3, 95 % CI 0·21, 0·80, respectively). Inverse associations with BC risk were also observed with the animal protein food group in overall and especially in postmenopausal women (OR=0·6, 95 % CI 0·40, 0·96 and OR=0·5, 95 % CI 0·28, 0·99, respectively).

**Table 3:** Association between food groups and breast cancer risk in cases and controls for daily median energy intake (highest *v.* lowest intake)* (Odds ratios and 95 % confidence intervals)

	Model A (*n* 792)			Model B (*n* 792)			ER^+^ † (*n* 312)			ER^–^ † (*n* 84)			Premenopausal (*n* 276)‡			Postmenopausal (*n* 516)‡		
Food groups	OR	95 % CI	*P*	OR	95 % CI	*P*	OR	95 % CI	*P*	OR	95 % CI	*P*	OR	95 % CI	*P*	OR	95 % CI	*P*
Cooked porridge (1518 kJ)	1·0	0·76, 1·40	0·859	0·9	0·73, 1·46	0·939	2·2	0·72, 6·92	0·173	0·9	0·62, 1·44	0·750	0·6	0·31, 1·34	0·183	1·2	0·82, 1·91	0·328
Starchy grains (2655 kJ)	1·3	0·88, 1·97	0·580	1·3	0·86, 2·08	0·222	0·6	0·13, 2·71	0·521	1·5	0·84, 2·45	0·153	0·8	0·33, 2·07	0·604	1·5	0·82, 2·84	0·154
Vegetables (275 kJ)	0·8	0·60, 1·29	0·447	0·9	0·64, 1·47	0·644	2·2	0·66, 8·64	0·265	0·8	0·53, 1·42	0·511	0·4	0·16, 1·28	0·090	1·2	0·76, 2·05	0·468
Fresh fruit (673 kJ)	0·6	0·45, 0·94	0·004	0·6	0·43, 0·94	0·022	0·8	0·13, 4·54	0·812	0·7	0·41, 1·12	0·108	0·3	0·12, 0·80	0·026	0·7	0·44, 1·23	0·164
Legumes (56 kJ)	0·9	0·73, 1·34	0·501	1·0	0·78, 1·47	0·872	0·5	0·14, 2·05	0·326	1·1	0·74, 1·76	0·488	1·9	0·93, 4·22	0·083	0·8	0·57, 1·35	0·344
Nuts and seeds (104 kJ)	1·0	0·74, 1·37	0·355	1·1	0·85, 1·52	0·609	0·2	0·58, 0·86	0·029	1·3	0·94, 1·82	0·249	1·1	0·52, 2·31	0·735	1·3	0·84, 2·04	0·323
Milk and milk products (327 kJ)	0·7	0·55, 1·03	0·937	0·8	0·61, 1·18	0·195	0·9	0·23, 3·82	0·951	0·8	0·51, 1·13	0·152	0·7	0·35, 1·66	0·449	0·8	0·53, 1·22	0·224
Animal protein (1728kJ)	0·6	0·41, 0·95	<0·001	0·6	0·40, 0·96	0·040	1·0	0·95, 1·01	0·475	0·6	0·35, 0·98	0·045	0·6	0·37, 1·68	0·351	0·4	0·28, 0·99	0·029
Fats and oils (453 kJ)	0·7	0·52, 1·02	0·004	0·7	0·52, 1·02	·068	0·3	0·11, 1·14	0·068	0·8	0·54, 1·33	0·385	0·5	0·28, 1·19	0·089	0·8	0·53, 1·44	0·470
Sugar (1668kJ)	0·8	0·57, 1·20	0·134	0·9	0·62, 1·48	0·667	1·3	0·34, 4·92	0·731	0·8	0·52, 1·37	0·460	1·9	0·86, 4·25	0·135	0·7	0·42, 1·37	0·299
Savoury snacks (388 kJ)	1·5	1·02, 2·30	0·292	1·9	1·12, 2·43	0·027	3·4	0·52, 24·32	0·216	1·5	0·91, 2·52	0·098	1·5	0·64, 4·22	0·389	2·1	1·15, 4·07	0·017
Animal protein§																		
Red meat (190 kJ)	0·6	0·43, 0·78	<0·001	0·6	0·49, 0·94	0·029	2·0	0·51, 8·52	0·342	0·6	0·38, 0·86	0·009	1·4	0·66, 3·35	0·384	0·5	0·32, 0·88	0·018
Organ meat (135 kJ)	0·7	0·50, 0·88	0·005	0·6	0·47, 0·91	0·022	1·8	0·43, 7·82	0·411	0·6	0·42, 0·91	0·014	0·6	0·33, 1·21	0·144	0·8	0·56, 1·28	0·284
Processed meat (316 kJ)	0·7	0·52, 0·94	0·012	0·9	0·61, 1·35	0·557	0·9	0·19, 4·10	0·885	0·9	0·57, 1·42	0·685	0·3	0·21, 1·02	0·071	1·1	0·68, 1·96	0·750
Fish (154 kJ)	1·1	0·94, 1·00	0·280	0·7	0·56, 1·17	0·130	0·5	0·10, 2·41	0·388	0·7	0·44, 1·14	0·162	1·0	0·96, 1·17	0·934	0·7	0·45, 1·14	0·121
Chicken (526 kJ)	0·6	0·56, 0·97	0·009	0·8	0·54, 1·25	0·281	0·5	0·08, 3·16	0·474	0·6	0·40, 1·05	0·080	0·4	0·29, 1·15	0·075	0·8	0·46, 1·47	0·374
Eggs (208 kJ)	0·9	0·92, 1·04	0·093	1·0	0·95, 1·09	0·187	0·9	0·96, 1·02	0·434	0·8	0·53, 1·32	0·410	0·9	0·86, 1·27	0·637	1·0	0·91, 1·02	0·418

ER^+^, oestrogen receptor positive, ER^–^, oestrogen receptor negative.

* Model A: crude output. Model B: adjusted for menopausal status, ethnicity, waist circumference, physical activity, level of education, income/month, use of birth control (hormonal/oral contraceptives), ethnicity, ever breast-feeding, age at menarche, age of menopause onset and family history of breast cancer.

† Stratified by ER tumour type.

‡ Stratified by menopausal status.

§ Break down of original animal protein compilation.

Additional analyses within the animal protein food group indicated that the subgroup ‘organ meat’ showed a significant inverse association with BC risk in fully adjusted model (OR=0·6, 95 % CI 0·49, 0·93). ‘Red meat’ showed a significant inverse association with BC risk in all women (OR=0·6, 95 % CI 0·49, 0·94) and especially in postmenopausal women (OR=0·5, 95 % CI 0·32, 0·88). Other subgroups in the animal food group (processed meat, fish, chicken and eggs) did not show any significant associations with BC risk.

After adjustment for confounders, savoury snack consumption showed a significant increased BC risk (OR=1·9, 95 % CI 1·12, 2·43), especially in postmenopausal women (OR=2·1, 95 % CI 1·15, 4·07). In addition, when exploring the association between BC risk and cancer subtypes, ER^–^ tumour showed an inverse association with animal protein consumption (OR=0·6, 95 % CI 0·35, 0·98), whilst ER^+^ stratification showed an inverse association of BC risk with consumption of nuts and seeds (OR=0·2, 95 % CI 0·58, 0·86).

## Discussion

This study aimed to investigate the association between dietary intake and BC risk in black South African women. We found an inverse association with BC risk and consumption of fresh fruit in premenopausal women, whilst subgroups of animal protein (red and organ meat) also showed inverse associations with BC risk. Savoury food consumption showed an increased BC risk in postmenopausal women. In addition, four out of five participants were either overweight or obese in both case and control participants.

Little attention is drawn to savoury foods in relation to BC risk possibly because savoury foods are more often associated with increased risk of gastric cancer^(^ ^27^ ^)^. A case–control and cohort studies from various populations found no association with added salt or spices and BC risk^(^ ^28^ ^,^ ^29^ ^)^. However, results from this study showed a strong increased BC risk with high savoury food consumption in model B and in postmenopausal women. A possible reason for this positive association with BC risk might be due to a combination of the high salt content, processed preparation methods of soups, sauces and potato crisps, lack of anti-oxidants and phytochemicals (previously proven to reduce BC risk^(^ ^4^ ^,^ ^30^ ^)^) and high total energy content in this food group.

Unexpectedly, the results of our study showed animal protein to be inversely associated with BC risk. The CUP report on BC states that there is limited evidence with no conclusions regarding an increased or decreased BC risk and consumption of animal protein^(^ ^4^ ^)^. Following on our findings, further analysis indicated that subgroups of animal protein, ‘red and organ meat’, showed inverse associations in women overall and with ER^–^ tumour subtype. When results were stratified by menopausal status, only ‘red meat’ showed an inverse association with BC risk in postmenopausal women.

Evidence investigating the association of BC risk with red meat and organ meat (as food groups) is lacking as consumption of organ meat in other countries may not be as much as in South Africa^(^ ^31^ ^)^. In South Africa red meat intake entails consumption of mostly organ and offal meat, as mutton, lamb and beef are mostly unaffordable for a large proportion of the South African population^(^ ^32^ ^)^. This was clearly observed in the information/data collected from the QFFQ.

Organ meat is less energy-dense and a more nutrient-rich protein (compared with red meat) and may contribute to explaining this inverse association with BC risk in black South African women. Red meat, however, is higher in energy due to a higher fat content and may increase the risk for obesity if over consumed. This is alarming since obesity is a known BC risk factor in postmenopausal women^(^ ^4^ ^)^. Furthermore, consumption of high amounts of red meat (120 g/d) has previously been linked to an increased risk of other cancers (colorectal, lung and prostate) in various populations^(^ ^33^ ^)^. Hence, the WCRF and American Institute for Cancer Research recommends limitation of red meat consumption (<500 g cooked weight per week) as part of a cancer prevention diet^(^ ^12^ ^)^. Epidemiological evidence for an increased BC risk with red meat consumption remains inconclusive but is suggestive of an increased BC risk^(^ ^33^ ^–^ ^35^ ^)^.

This study population had a low consumption of red and organ meat (total animal protein intake in cases accounted for <31 g/d and <2 % of total energy intake) and may contribute to the observed inverse association with BC risk. This in turn can also be attributed to recall bias and high rates (49 %) of late stage BC (stage III/IV), diagnosis. During late stage BC, patients often present with decreased appetite and altered taste acuity (dysgeusia, hypogeusia and ageusia) and may influence habitual dietary intake^(^ ^36^ ^)^. Furthermore, given the case–control study design of this study, reverse causality is likely to occur. The interpretation of the results presented above are further complicated by the coding methods currently used in South Africa and will be discussed furthermore in the limitation section. A number of factors mentioned above may influence the association between BC risk and red and organ meat consumption in this specific population. Therefore, this inverse association with red and organ meat requires further investigation as no other case–control study has found an inverse association with BC risk.

Less energy-dense foods such as fruits and vegetables are often associated with a decreased BC risk and is recommended by the WCRF and South African FBDG for prevention of non-communicable diseases^(^ ^12^ ^,^ ^37^ ^)^. These foods are lower in energy and rich in nutrients which contribute to maintaining a healthy body weight. Antioxidants and phytochemicals present in these less energy-dense foods (fruit and vegetables) have also shown to reduce BC risk^(^ ^30^ ^)^. The CUP report states that there is currently limited evidence of a significant decrease in BC risk associated with non-starchy vegetables and other less energy-dense food groups^(^ ^4^ ^)^. In this sample, a significant protective association for decreased BC risk was found with higher fruit consumption (673 kJ/ more than 1½ fruit servings/d) in ER^–^ and premenopausal women. No significant associations were found for other less energy-dense food groups such as vegetables. However, the portion sizes of vegetables eaten are usually very small. In this sample, vegetables accounted for 3 % of the total energy intake in both case and control participants and may be an indication that the recommendation of the SAFBDG of 400 g vegetables/d (together with fruit) was not met^(^ ^13^ ^)^.

It is therefore important to investigate the dietary intake in this sample as a whole. Energy-dense foods from four food groups (cooked porridge, starchy grains, sugar and animal protein) accounted for more than 75 % of the total energy intake, whilst less energy-dense food groups represented <18·5 % in case and control participants. Dietary intake from this study is therefore in line with previous research on dietary intake in the black female population of South Africa where a monotonous diet associated with a Westernised diet was noted^(^ ^12^ ^–^ ^15^ ^)^. In South Africa, more energy-dense staple foods such as maize meal and bread are fortified with micro-nutrients. However, it is not known whether fortified food improve nutritional status of black South African women^(^ ^38^ ^)^. Thus, high consumption of mostly energy-dense foods (generally also nutrient-poor foods) in this sample is bothersome for a diet consisting mainly out of energy-dense and nutrient-poor foods and is not nutritionally adequate for optimal health^(^ ^37^ ^)^ and prevention of BC.

Not all energy-dense foods are unhealthy, but overconsumption of more energy-dense foods may lead to a higher total energy intake/d, increasing the risk of obesity that is a known BC risk factor in postmenopausal women. Worrisome overweight and obesity rates mentioned above, together with high total energy intake per d and low physical activity, were noted in this study. Attention is thus also drawn to obesity as a possible risk factor that may contribute to increased BC risk in black South African women.

Moreover, it is acknowledged that healthier, less energy-dense foods in South Africa are costlier than unhealthier more energy-dense foods (mostly refined grains) such as cooked porridge, starchy grains, sugar, processed meat and fats^(^ ^32^ ^,^ ^38^ ^)^. Previous studies stated that dietary intake was directly linked to income in a social–economic-restricted environment^(^ ^38^ ^)^. A low-income distribution was noted in this study where 85·8 % of the sample earned less than R3001·00/month. Poverty may therefore contribute to lower intake of healthier less energy-dense and nutrient-rich foods that may protect against BC.

Control participants had a higher total energy consumption in almost all food groups and in total energy intake compared with case participants. Since control participants had a higher BMI and waist circumference, these results were expected. Lower energy intake seen in case participants may be due to delayed BC diagnoses and altered dietary intake in case participants.

### Limitations

The sample size of this study was small, however, data regarding diet and BC risk are lacking in South Africa. Therefore, results of this study are indicative of much needed data on dietary intake and BC risk in black South African women. Coding of single foods using the South African food composition tables may have contributed to inaccurate grouping of foods within food groups. Some meals (stews), consisting out of two food groups (animal protein and starchy grains), were coded as a single food when in fact it could have been two separate single foods divided in different food groups. Underreporting of certain foods is also a limitation since dietary data are just an estimation of dietary intake and relies on the subject’s memory. In addition, this study did not collect data on some risk factors associated with BC risk such as genetic mutations and time period between participants’ last breast-feeding period and BC diagnoses.

### Strengths

This study had a population-based and matched case–control study design which improved statistical precision. This study provided much needed evidence for an African population group in relation with BC risk since data on this topic are lacking. All questionnaires used to collect data were proven to be validated and data used in the analysis were highly standardised. All case participants were recruited before any BC treatment.

In conclusion, consumption of fresh fruit, red and organ meat showed an inverse association with BC risk, whilst savoury food consumption showed an increased BC risk. However, no other studies have found an inverse association with BC risk and red or organ meat consumption. Therefore, further research is necessary to investigate the association with BC risk and red and organ meat in black South African women. Moreover, dietary intake in this sample were in line with a westernised diet, whilst alarming overweight and obesity rates together with low physical activity levels were noted in this sample. A diet with foods lower in energy and higher in nutrients such as fresh fruit and vegetables in combination with a decrease consumption of energy dense foods like savoury foods are advised as a possible preventative diet for BC and strategy to reduce bothersome obesity rates in black South African women. However, poverty influences food choices, and health interventions in South Africa should strive to implement affordable and accessible methods in line with these recommendations.
