# Supplementary material for: Dietary intake and breast cancer risk in black South African women: the South African Breast Cancer study
Source: Br J Nutr. 2019 Feb 1;121(5):591–600. doi: 10.1017/S0007114518003744 (PMC6521785; doi:10.1017/S0007114518003744)
Supplement: Supplementary file 1 [file S0007114518003744sup001.docx]

**Supplementary Figure 1**. Percentage of total median energy intake distribution in food groups.

*Animal food group compilation
